# Examining the cooperation between extrachromosomal DNA circles

**DOI:** 10.7554/eLife.84639

**Published:** 2022-12-07

**Authors:** Jiahui Zhang, Cheng-Zhong Zhang

**Affiliations:** 1 Department of Data Science, Dana-Farber Cancer Institute Boston United States; 2 Department of Biomedical Informatics, Blavatnik Institute, Harvard Medical School Boston United States; 3 Cancer Program, Broad Institute of MIT and Harvard Cambridge United States

**Keywords:** extrachromosomal DNA, oncogenes, super-resolution imaging, transcription factors hub, glioblastoma, cancer, Human

## Abstract

In a departure from previous findings, new results suggest that free-floating pieces of DNA which carry additional copies of cancer-driving genes do not tend to cluster or have increased transcription.

**Related research article** Purshouse K, Friman ET, Boyle S, Dewari PS, Grant V, Hamdan A, Morrison GM, Brennan PM, Beentjes SV, Pollard SM, Bickmore WA. 2022. Oncogene expression from extrachromosomal DNA is driven by copy number amplification and does not require spatial clustering in glioblastoma stem cells. *eLife* **11**:e80207. doi: 10.7554/eLife.80207.

Cells often need to modulate the production of certain proteins to adjust to their ever-changing environment. This is usually achieved by altering the amount of messenger RNAs synthetized from the corresponding genes. Two factors impact the transcription yield of a gene: the number of active copies of this sequence in the genome, and the rate at which each of them is transcribed.

Boosting the production of a protein is usually achieved by increasing transcription rates, but special cases can involve directly creating more copies of the associated gene (Stark and Wahl, 1984). This phenomenon was first identified in amphibian eggs, where it helps cells to produce the elements required for protein synthesis (see Tobler, 1975 for review). In human cells, however, gene amplification is most commonly associated with boosting the expression of cancer-driving genes (Tanaka and Watanabe, 2020). These additional ‘oncogene’ copies can be arranged in tandem in a specific region of a linear chromosome, or they can be contained inside small circles of extrachromosomal DNA (ecDNA) formerly known as double-minute chromosomes (Verhaak et al., 2019; Cox et al., 1965; Figure 1A). A recent study by Hung et al. has reported that oncogenic ecDNAs frequently come together to form hubs of 10 to 100 ecDNA circles; inside these clusters, intermolecular interactions take place that boost oncogene expression (Hung et al., 2021). Now, in eLife, Steven Pollard, Wendy Bickmore and colleagues at the University of Edinburgh — including Karin Purshouse as first author — report new results which contradict these findings (Purshouse et al., 2022).

**Figure 1.**
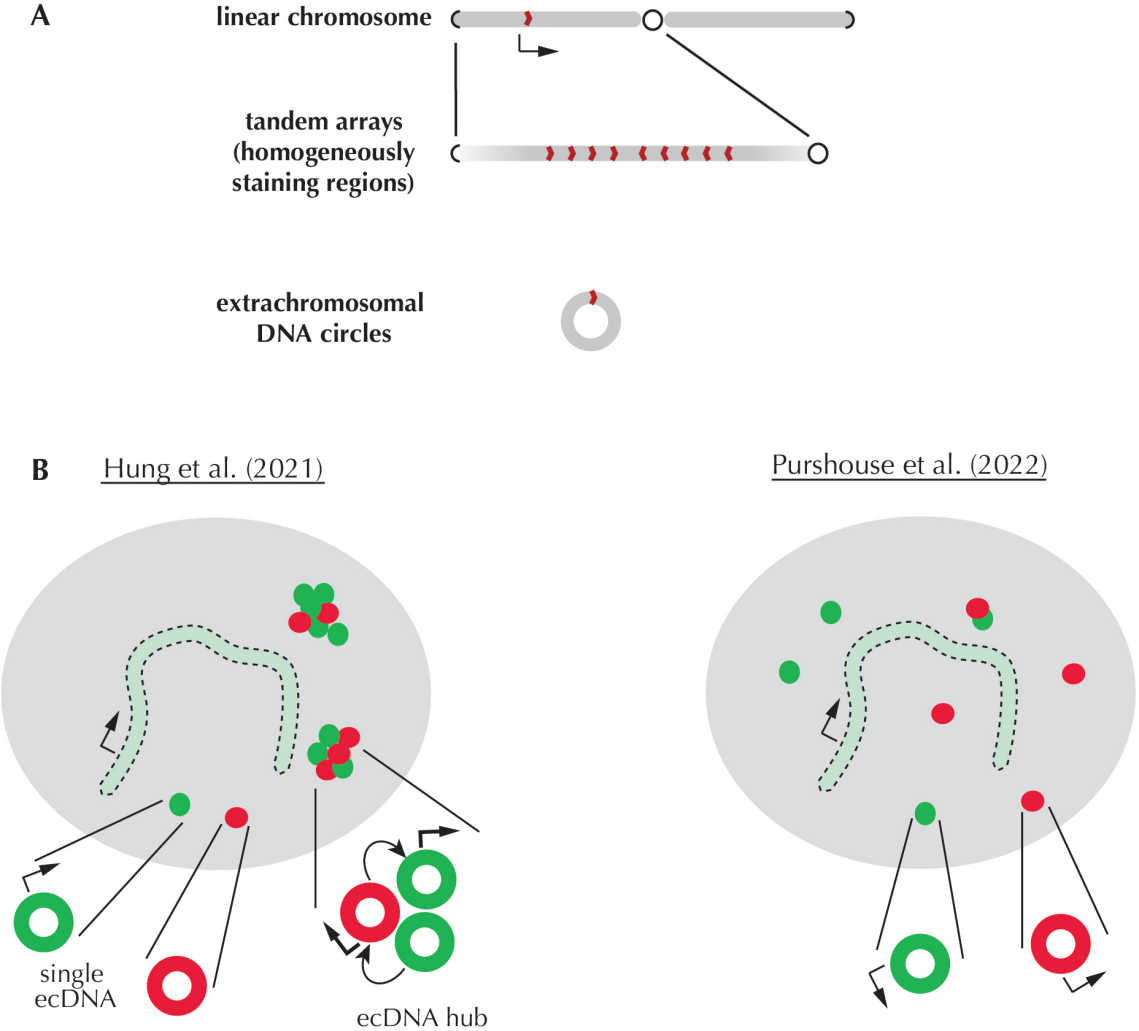
Extrachromosomal DNA circles carrying cancer genes do not tend to cluster and be transcribed more efficiently. (**A**) A linear chromosome (top) is ‘book-ended’ by telomeres (repeat sequences that that prevent fusion between chromosome ends; represented as black ‘bracket-like’ shapes). It also displays a constricted region known as the centromere (black circle), which allows chromosomes to segregate properly during division. An oncogene (red shape pointing to the right) is normally present in one transcribed (arrow) copy on a linear chromosome. In cancer cells, oncogenes can be amplified to form tandem arrays (middle) that create aberrant chromosomal segments known as homogeneously staining regions. Individual oncogene copies in these regions may be arranged at both orientations (as defined by how these sequences will be read by the transcription machinery; red shapes pointing to the left or right). Additional oncogene copies can also be carried by small circular DNA structures without centromeres or telomeres (bottom), which are known as extrachromosomal DNA (ecDNA) circles. As ecDNAs do not contain centromeres, they often segregate improperly or even fuse together; this causes one of the daughter cells to inherit more amplified ecDNA circles, and over time, to accumulate many oncogene copies. (**B**) A 2021 study by Hung et al. (left) showed that amplified ecDNA circles carrying various oncogenes (red and green) often come together to form hubs. These clusters enable intermolecular interactions (curved arrows) that boost transcription (thicker arrows), with oncogene copies carried by isolated ecDNA and the chromosome being less transcribed. In contrast, Purshouse et al. did not observe oncogenic ecDNA clusters in glioblastoma cells; in addition, their analyses revealed that ecDNA and chromosomal oncogene copies were transcribed at the same rate. These diverging results may stem from the two teams having used different types of cancer cells for their experiments, highlighting the need for further research.

The team focused on malignant cells from an aggressive type of brain cancer known as glioblastoma; these frequently contain ecDNA circles carrying one or multiple oncogenes. Using super-resolution imaging, Purshouse et al. were able to determine the location of individual ecDNA circles within the nucleus, and how frequently two ecDNAs were found within a given distance. Comparing these numbers with what would be expected if the circles were randomly distributed in the nucleus allowed the team to assess whether ecDNAs form clusters more frequently than anticipated. When analyzing ecDNAs carrying the same oncogene, they found no evidence of clustering of ecDNAs within 200 nm – the distance that corresponds to the estimated diameter of ecDNA hubs.

However, due to the limitation of optical resolution, this approach cannot detect tighter clusters consisting of ecDNAs that are closer than 200nm. To overcome this challenge, Purshouse et al. used another line of glioblastoma cells that carry two distinct types of ecDNAs, which were imaged independently using different probes. This approach makes it possible to spot smaller hubs that bring together different ecDNA ‘species’, yet it also did not provide evidence that ecDNAs cluster in glioblastoma cells.

The team then focused on how ecDNA circles were being transcribed. First, they examined whether oncogenic ecDNAs may be clustering with ‘transcriptional hubs’ that physically bring together various elements of the transcription machinery. However, no spatial correlation was found between ecDNAs and these hubs. Next, they compared how chromosomal and ecDNA copies of the same oncogene were being transcribed. They started by imaging nascent RNA transcripts near individual ecDNA circles, gathering information that allowed them to assess the fraction of ecDNAs that are being actively transcribed at any given time. These analyses captured ‘immature’ RNA transcripts as they were being synthesized, and they showed that ecDNA and chromosomal copies were transcribed at a similar frequency.

Finally, the team switched their focus to mature messenger RNAs. They estimated the fraction of messenger RNAs transcribed from either the ecDNA or the chromosomal copy of an oncogene, using slight differences in the sequences between the two versions. After normalizing for ecDNA and chromosomal copy numbers, they established that an individual sequence, whether chromosomal or extrachromosomal, would produce a similar amount of mature messenger RNAs. Chromosomal DNA and ecDNA therefore appear to be transcribed with a similar efficiency. Taken together, these findings suggest that the transcriptional yield of amplified ecDNAs is primarily determined by the number of ecDNA circles, rather than the cooperative transcription of clustered ecDNAs.

What could explain the discrepancy between these observations and the results reported by Hung et al. (Figure 1B)? Purshouse et al. suggested that both sets of conclusions may in fact be true, but under different circumstances. As these studies relied on a small number of cell lines derived from different types of tumors, diverging results could reflect variations in the size, gene composition or copy number of ecDNAs across cancers. In addition, ecDNAs are highly dynamic; they can recombine, reintegrate within a chromosome or undergo other types of molecular rearrangements which may all alter transcription kinetics (Shoshani et al., 2021; Rosswog et al., 2021). Further studies are now needed to explore the way that ecDNA transcription changes across a wide range of cancers and during disease progression.
